# Benefit of COVID-19 vaccination accounting for potential risk compensation

**DOI:** 10.1038/s41541-021-00362-z

**Published:** 2021-08-11

**Authors:** John P. A. Ioannidis

**Affiliations:** Departments of Medicine, of Epidemiology and Population Health, of Biomedical Data Science, and of Statistics, and Meta-Research Innovation Center at Stanford (METRICS), Stanford University, Stanford, CA USA

**Keywords:** Medical research, Immunology

## Abstract

People receiving COVID-19 vaccines may subsequently markedly increase their previously suppressed exposure risk. A simple model can evaluate the benefit of vaccination to the vaccinated (index) person and others exposed to that person; and calculate the amount of risk compensation required to eliminate all the benefits or to halve the benefit. As shown, 2.5-fold increase in exposure will eliminate the benefit of a vaccine of moderate efficacy (*E* = 0.6) unless the probability of infection in the population of interest is very high. With very high vaccine efficacy (*E* = 0.95), substantial benefit is maintained except in situations where there is a very low probability of infection in the population. If the vaccine efficacy decreases to 0.8, the benefit gets eroded easily with modest risk compensation. Risk compensation may markedly affect the benefit of COVID-19 vaccination, especially if vaccine efficacy in real-life or specific high-risk populations (e.g., nursing home residents) is not very high.

## Introduction

Vaccines are a stellar achievement of public health and the development of COVID-19 vaccines has been characterized as the top scientific development of 2020^1^. Phase 3 efficacy data on several COVID-19 vaccines are now available, with some^2,3^ (but not all)^4^ of them exceeding 90% reduction in documented infections and a favorable safety profile. Rapid deployment has tried to reach fast as many people as possible, with priority given to high-risk and high-exposure groups. Hopefully, COVID-19 vaccines may save lives through reducing the risk of infection among vaccinated individuals. Additional postulated benefits may include a slowing of epidemic waves—and possibly even their closure through herd immunity.

COVID-19 vaccines may help regain some normality in other aspects of life at the private and societal levels. Normal activities are affected by various nonpharmaceutical measures that try to diminish the spread of the virus, e.g. distancing interventions and avoidance of exposures^5^. However, could easing of these measures and changes in behavior after vaccination erode the benefit of vaccination by an increase in the infection risk? This possibility is part of the broader concept of risk compensation (aka danger compensation or risk homeostasis)^6–9^. According to this concept, individuals who receive preventive interventions may increase their risky behavior, willing to handle higher risk exposures given the protection they have received. In the case of COVID-19 vaccination, this would mean that vaccinated individuals may start meeting with more people and expose themselves to situations of higher infection risk. They may also be more willing to meet people at high risk of serious outcomes upon infection. For people who are largely dependent on caregivers for their choices, e.g., nursing home residents, caregivers may consciously or unconsciously put these dependent people at higher risk, reassured from the protection of vaccination.

Risk compensation is a debated theory and some researchers argue that empirical data have not upheld it as a major threat in the real-world^10,11^. The debate has surrounded areas as diverse as the use of seat belts and helmets for driving, pre-exposure prophylaxis and circumcision to prevent HIV infection, and HPV vaccination to prevent cervical cancer^10^. For COVID-19, the concept of risk compensation was already debated in the case of face coverings^10^. The dominant view has been that face coverings do not increase risky behavior, and may even provide cues to diminish risky exposure. However, behavior change can be complex and must be carefully gauged to maximize benefits^12^. Different circumstances may lead to different outcomes^13^.

Here, I present a simple modeling approach to try to understand how risk compensation may or may not affect the overall benefit of vaccines for COVID-19. The model can be generalized to other situations of different preventive measures with risk compensation. However, specific characteristics peculiar to COVID-19, its transmission, spread, and exposure settings make the COVID-19 application particularly interesting.

The model considers the risk of death, as the most severe outcome of COVID-19 infection, but similar considerations can be made also for other outcomes that matter (e.g., severe disease, need for hospitalization, or need for admission to the ICU) or even for simple infection. Vaccine efficacy may vary for different outcomes and risk compensation may thus have different connotations for them.

The model also considers two main components of risk: the risk to the index person being vaccinated, and the risk imposed to others by the index person. Both of these components of risk may change as behavior changes post-vaccination. Behavior may change because of personal choice and/or due to changes in mandates by public health and other authorities that may modify their stance as more people get vaccinated.

## Results

### Risk compensation required to eliminate benefit

**Fig. 1:**
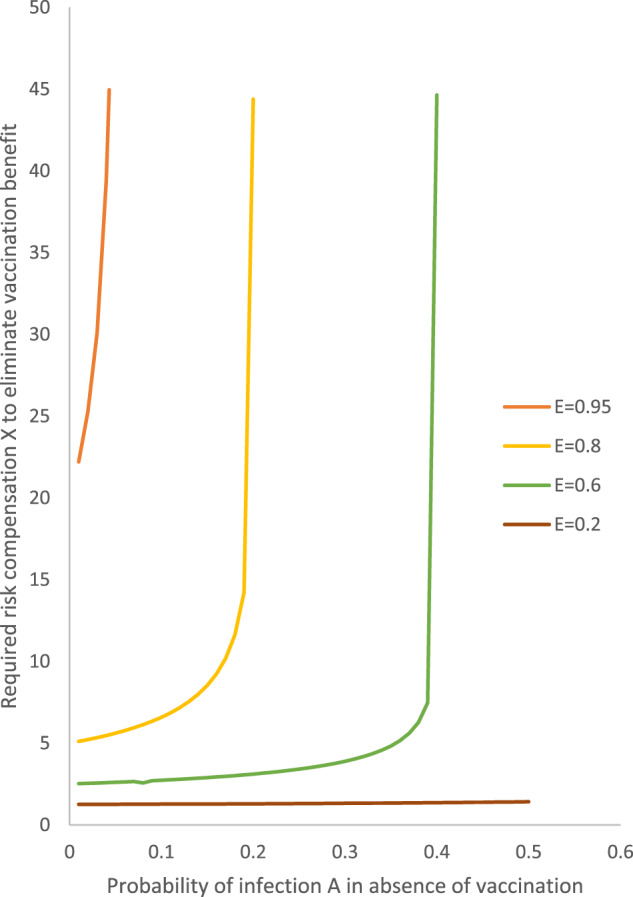
Required risk compensation to eliminate the vaccination benefit. Scenarios presented with vaccine efficacy (E) of 0.2, 0.6, 0.8, and 0.95.

If vaccination leads to *X* fold increased exposure load due to risk compensation, then, as shown in Fig. 1, the required risk compensation *X* to eliminate the vaccination benefit increases very sharply towards infinity as the probability of infection A approaches 1 − *E*, where *E* is the efficacy of the vaccine. Of note, the benefit cannot be entirely eliminated when the probability of infection A is 1 − *E* or higher.

For low values of probability of infection A (<3%), the required risk compensation to eliminate the vaccination benefit is very high (20–30-fold) for a vaccine with very high efficacy (*E* = 0.95). However, it is modest (approximately fivefold) for a vaccine with high efficacy (*E* = 0.8) and it is very low for vaccines with moderate efficacy (only 2.5-fold for *E* = 0.6) or low efficacy (barely 1.25-fold for *E* = 0.2).

With higher probabilities of infection A in the range of 3–30%, the required risk compensation remains very low when vaccine efficacy is 0.6 or less. With probabilities of infection A in the range of 3–18%, the required risk compensation to eliminate all benefits is modest (<10-fold) even for a vaccine with high efficacy (*E* = 0.8) for A < 10%.

### Risk compensation required to halve benefit

**Fig. 2:**
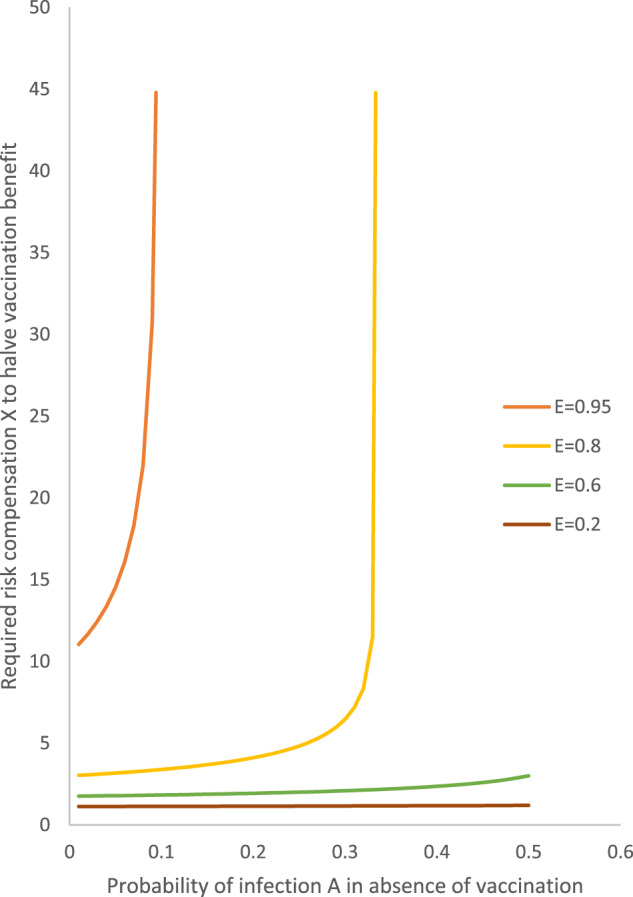
Required risk compensation to halve the vaccination benefit. Scenarios presented with vaccine efficacy (E) of 0.2, 0.6, 0.8, and 0.95.

As shown in Fig. 2, the required risk compensation *X* to halve the vaccination benefit increases very sharply towards infinity as *A* approaches 0.10 for *E* = 0.95 and as *A* approaches 1/3 for *E* = 0.80. Above these levels of A, the benefit cannot be halved for vaccines of such very high or high efficacy, respectively.

With the probability of infection <5%, the required risk compensation to have the benefit is 10–15-fold with very high vaccine efficacy (*E* = 0.95), but it is low even with high efficacy (<3.2-fold for *E* = 0.80) and it is very low with *E* = 0.60 (<1.8-fold) and negligible with *E* = 0.20 (<1.3-fold).

For vaccines with high efficacy (*E* = 0.80) one does not reach risk compensation values exceeding 5, except at high probabilities of infection (*A* > 25%) and *X* > 10% in seen only with *A* > 32%, very close to the infinity threshold of *A* = 33.3%. For vaccines of moderate or low efficacy, values of the required risk compensation to halve the vaccination benefit remain low or negligible, respectively, even with very high values of A.

### Calculations with impact of vaccination on infection fatality rate

When an index is infected despite vaccination, if the vaccination diminishes the infection fatality rate to half (*H* = 0.5), then for a highly effective vaccine (*E* = 0.95) the risk compensation R to halve the benefit reaches infinity as early as *A* = 5% and it impossible to halve the benefit for higher values of *A*. With *H* = 0.5, with *E* = 0.8, the *R* to halve the benefit is 5–10 for values of *A* up to 14% and reaches infinity at *A* = 18.1%.

In the scenario where *H* = 2 (doubled infection fatality rate for an index infected despite vaccination), even with *E* = 0.95 the risk compensation *R* required to halve the benefit remains below 10 for values of *A* up to 14%; with *E* = 8 the *R* required to halve the benefit remains consistently very low, barely reaching 3 for *A* = 50%.

## Discussion

Risk compensation needs to be factored carefully when appraising COVID-19 vaccination strategies. The simplified model used here suggests that even negligible risk compensation can eliminate the benefit of a vaccine of low efficacy, and it also takes only 2.5-fold increase in exposures for the benefit of a vaccine of moderate efficacy (*E* = 0.6) to disappear unless the probability of infection in the population of interest is very high. With very high vaccine efficacy (*E* = 0.95), the substantial benefit is guaranteed except in situations where there is a very low probability of infection in the population, in which case sufficiently large risk compensation can even eliminate whatever benefit is conferred. If the vaccine efficacy is even eroded minimally from 0.95 to 0.8, the benefit gets eroded easily and it disappears even with modest risk compensation, unless the probability of infection in the relevant population is high.

As of this writing (late December 2020), some COVID-19 vaccines (the ones marketed by Pfizer and Moderna)^2,3^ seem to fit the pattern of very high efficacy based on phase 3 data with limited follow-up, while others (like the one by AstraZeneca)^4^ seem to have moderate to high efficacy. However, some vaccines may see their efficacy further eroded with longer follow-up and/or as new variants of SARS-CoV-2 arise and become dominant in various populations. Based on the presented simulations, a vaccine with moderate efficacy may be a poor choice when even very modest risk compensation is likely to happen. The exact extent of risk compensation that will ensue upon COVID-19 vaccination needs to be carefully studied with empirical data. In other fields where risk compensation has been discussed in the past (high-risk driving and sexual behavior), the levels of risk compensation seem to have been low^10,14^; some data suggest even an inverse phenomenon (where those exposed to the preventive intervention even have lower levels of risky behavior^14^, but this may be a spurious observation due to confounding (those who use the preventive measure are more health-conscious). With COVID-19 vaccination, the level of risk compensation may be much higher than for other public health problems where risk compensation has been debated. The reason is that COVID-19 has already led to a marked reduction in exposure risk trying to contain the pandemic. The adopted suppressive behaviors, largely centered around social distancing, are highly strenuous. The hope is that vaccination should lead to regained normality, i.e. relieving these extreme suppressive behaviors. In high-risk settings for COVID-19 mortality like nursing homes, the existing reduction in exposures may be even larger than in other settings.

Vaccines with documented very high efficacy appear to withstand the impact of even large risk compensation. However, it is important to validate whether efficacy seen in phase 3 trials also translates into similar and lasting efficacy and effectiveness for hard outcomes in the real-world^15,16^, and even more so in specific high-risk settings such as nursing homes that were not assessed in phase 3 trials. Real-world data as of spring 2021 are very favorable, but they need to continue to be collected and carefully analyzed for all vaccines and also re-assessed as new variants may emerge and become dominant in various locations around the world. Phase 3 trials have suggested a similar efficacy in elderly individuals, but these were largely community-dwelling elderly^2,3^. It needs to be validated whether nursing homes residents who are typically far more debilitated than community-dwelling elderly clinical trial participants can also launch a similar immune response. If not, even slight compromise of the efficacy from 0.95 to 0.8, can make the challenge of risk compensation highly relevant. Similar challenges may exist for people with immune suppression who may not mount sufficient immune response.

A practical corollary is that until we get more evidence on the real-world effectiveness of each authorized vaccine and in particular of their effectiveness in nursing homes and other high-risk settings, intensified precautions in these settings should continue. It may be best to wait for the epidemic wave to be largely suppressed before relaxing some existing strategies like repeated PCR testing in high-risk settings. In most western countries, nursing home fatalities have accounted for a lion’s share (30–70%) of all fatalities^17,18^. In the USA alone, at least 38% of deaths (over 120,000) and over 900,000 documented infections occurred in chronic care facilities as of late December 2020^19^. The continued accumulation of deaths in such facilities suggests that the measures taken to-date have not been bullet-proof. In fact, it is probable that in many western countries with high fatality rates, the proportion of people infected in nursing homes exceeded the proportion of people infected in the general, community-dwelling population^18^. It may be a wrong choice to diminish protective measures in these extremely high-risk settings trusting in vaccination alone. Conversely, while the epidemic wave in a given location is still active, measures like regular routine testing of all staff and residents should be intensified, and exposures should continue to be controlled. Paradoxically, over-trusting the security of vaccination could lead to an excess of deaths.

Given these considerations, it is important to generate quickly data on the efficacy of vaccination on nursing home residents and other high-risk groups typically prioritized in the vaccine deployment schedules. This should include studies of immune response (and its durability and relevance for emerging variants) after vaccination in previously under-studied groups; collection of data on any infections and COVID-19 deaths after vaccination in these groups;^20^ and examination of the proportion of COVID-19 fatalities contributed by these groups and of any excess mortality trends within these groups.

The presented framework also gives insights on the relative contributions of vaccination to a personal benefit versus a benefit to others. Of note, for *H* = 1, the ratio of benefit to index and benefit to other people is always 1/Rs. For *R* = 1, the benefit to other people exceeds the benefit to the index by s-fold. The difference gets attenuated with lower *R* and accentuated with larger *R*. *R* may depend to a large extent on the proportion of immune people in the population where the index operates, with immunity shaped either by natural infection or by vaccinations. Of course, *A* and *R* are also inter-related and classic formulas exist for their relationship under simplified conditions^21,22^. However, their exact relationship may be far more complex in the presence of a vaccination intervention with heterogeneous population mix.

If the vaccination results in *H* < 1, i.e., partly protects people from death even if they get infected after vaccination, then more risk compensation could be tolerated without the vaccination benefit being lost. Conversely, if the vaccination results in *H* > 1, i.e., the people who get infected despite vaccination have a heightened infection fatality rate, then even less risk compensation would erode the vaccination benefit. Studies on the real-world impact of vaccination on mortality are needed to get better insights into this issue.

The modeling used here is simple, aimed to serve illustrative purposes for key aspects rather than capturing the full complexity of the problem. Previous work on risk compensation after vaccination has used more complex models, e.g., compartment models in the case of HIV vaccination^23–25^, also suggesting that HIV vaccination with a partially effective HIV vaccine can do more harm than good in the long term^24^. More complex models may also be developed for COVID-19, but they should take into account the inherent differences between a pathogen spreading fast in very acute epidemic waves versus a chronic epidemic like AIDS that spreads over decades.

Some other limitations should also be discussed. First, the presented modeling assumes that those who launch an immune response to vaccination will not be infected regardless of how massively and repeatedly they are exposed. In other words, the model considers *E* to be fixed regardless of the intensity of the exposure. This assumption may be invalid. If *E* also decreases with more massive exposure, then the amount of risk compensation needed to eliminate the vaccination benefit would also decrease. Second, there is evidence that a substantial amount of viral transmission with COVID-19 may happen through superspreader events^26^. Knowledge of the determinants of superspreader events may help gauge risk compensation, avoiding such risk compensation in settings that foster superspreader events and allowing for more laxity towards regaining normal life practices in settings where superspreader events are improbable. However, knowledge allowing high predictability of superspreader events is rather rudimentary to date. Third, the model does not factor the possibility of evolutionary pressure on the virus leading to mutations with lost vaccine effectiveness. This has been considered a problem induced by risk compensation in HIV infection under partially protective vaccines^25^. If true also to some extent for COVID-19, risk compensation may compromise vaccination benefits through an additional mechanism. Fourth, it is unknown whether some people may be less likely to be infected than others, despite having an equivalent amount of exposure. If so, the most susceptible may tend to be infected earlier and increasing levels of exposure will not infect as many more as expected by the (1 − (1 − *A*)^*X*^) factor in the model. If there is substantial inherent resistance to infection in many individuals, this would allow for higher risk compensation without erosion of the vaccination benefit.

Finally, the calculations presented here have focused on estimating values of *X* that lead to the elimination or halving of the benefit on a relative scale. However, unless entirely eliminated, the absolute magnitude of the risk is very important for decision-making purposes^27^. The absolute benefit depends on the epidemic activity (hence *A* and *R*) and the infection fatality rate of the index and other people. Not surprisingly, with low epidemic activity the benefit is low and given that it is also easily eroded by risk compensation, using COVID-19 vaccines in settings of low anticipated epidemic activity is unlikely to procure much benefit. Similarly, the benefit would be of very low magnitude in vaccinating low-risk people, e.g., children and very young adults, unless these low-risk people operate in settings where they infect primarily individuals with much higher infection fatality rate.

Allowing for these caveats, the present analysis provides insights about the possible impact of risk compensation. It also suggests the need to collect some additional pieces of evidence to inform better, more comprehensive models of risk compensation for COVID-19. Under no circumstances should this work be seen as questioning that COVID-19 vaccines are a stellar achievement with great potential, if used properly and strategically. The greatest benefit may be reaped in locations where the virus has not yet spread widely and remain at risk of high attack rates of new infections in the near future in the absence of protection from vaccination. However, to obtain optimal benefit and avoid harms, along with rapid deployment of these vaccines to as many vulnerable individuals as possible, a thoughtful vaccination strategy should be in place to minimize the potential impact of risk compensation.

## Methods

### Personal benefit of vaccination

In the absence of vaccination, the index personal risk of death is given by the product of the probability of infection A and the infection fatality rate f of the index, i.e.

1 $${{{D}}}_{{{{\mathrm{index,}}}}\;{{{\mathrm{no}}}}\;{{{\mathrm{vaccination}}}}} = {{{\mathrm{Af}}}}$$

With vaccination, the vaccine decreases the probability of infection to (1−*E*)-fold, where *E* is the efficacy of the vaccine. In the presence of risk compensation, the vaccination leads to *X* times higher exposure load. The increased exposure load aggregates the cumulative impact of meeting more people, for lengthier periods, and/or at higher risk circumstances, e.g., with less adherence to distancing, masks, and/or testing). The model assumes for simplicity that *E* percentage of indices would be immune and not possible to infect regardless of how much they are exposed, while the remaining 1 − *E* percentage (the infectable group) can be infected. It also assumes that each additional unit of exposure of equal magnitude to the baseline pre-vaccination exposure changes the chances of remaining uninfected in the infectable group by (1 − *A*)-fold. With *X*-fold exposure, the probability of not being infected for the infectable group thus becomes (1 − *A*)^*X*^, and the probability of being infected is 1 − (1 − *A*)^*X*^. It is unknown whether vaccination may affect also by *H*-fold the infection fatality rate for the index when/if that person does get infected despite having been vaccinated. Therefore, for an index person the personal risk of death with vaccination becomes

2 $${{{D}}}_{{{{\mathrm{index,}}}}\;{{{\mathrm{vaccination}}}}} = \left( {1 - {{{E}}}} \right)\left( {1 - \left( {1 - {{{A}}}} \right)^{{{X}}}} \right){{{\mathrm{fH}}}}$$

The net benefit for the index person is

3 $${{{\mathrm{Benefit}}}}\left( {{{{\mathrm{index}}}}} \right) = {{{D}}}_{{{{\mathrm{index,}}}}\;{{{\mathrm{no}}}}\;{{{\mathrm{vaccination}}}}} - {{{D}}}_{{{{\mathrm{index,}}}}\;{{{\mathrm{vaccination}}}}} = {{{\mathrm{Af}}}} - \left( {1 - {{{E}}}} \right)\left( {1 - \left( {1 - {{{A}}}} \right)^{{{X}}}} \right){{{\mathrm{fH}}}}$$

For this benefit to be eliminated because of risk compensation, one needs to have

*A*f − (1 − *E*)(1 − (1 − *A*)^X^)f*H* = 0, and solving for *X*, this becomes

4 $${{{X}}}\left( {{{{\mathrm{index,}}}}\;{{{\mathrm{eliminated}}}}\;{{{\mathrm{benefit}}}}} \right) = {{{\mathrm{log}}}}\left[ {\left( {{{{H}}} - {{{A}}} - {{{\mathrm{EH}}}}} \right)/\left( {{{{H}}} - {{{\mathrm{HE}}}}} \right)} \right]/{{{\mathrm{log}}}}(1 - {{{A}}})$$

while for *H* = 1, this is log[(1 − *A* − *E*)/(1 − *E*)]/log(1 − *A*)

For the benefit to be halved because of risk compensation, one needs to have

2(*A*f − (1 − *E*)(1 − (1 − *A*)^X^)f*H*) = *A*f − (1 − *E*)AfH, and solving for *X* this becomes

5 $${{{X}}}\left( {{{{\mathrm{index,}}}}\;{{{\mathrm{halved}}}}\;{{{\mathrm{benefit}}}}} \right) = \log \left[ {\left( {2 + {{{\mathrm{AE}}}} - {{{A}}} - \left( {{{{A/H}}}} \right) - 2{{{E}}}} \right)/\left( {2\left( {1 - {{{E}}}} \right)} \right)} \right]/\log (1 - {{{A}}})$$

while for *H* = 1, this is log[(2 + *AE* − 2*A* − 2*E*)/(2(1 − *E*))]/log(1 − *A*)

### Benefit of vaccination for other people

In the absence of vaccination of the index person, the risk imposed to other people reflects the product of the number of people R who would be infected by an infectious index person, the probability of infection of the index person A, and the infection fatality rate of the other (non-index) people to which the infection is transmitted. People exposed to and possibly infected from the index case may have a similar or very dissimilar infection fatality rate than the index case. E.g., if they all have a similar age and comorbidities profile (e.g., co-players at some sports team), then their infection fatality rate would be similar. Conversely, the infection fatality rate may be different, if the exposed have a very different profile than the index (e.g., debilitated nursing home residents exposed to an infected young, healthy staff member). If the infection fatality ratio in the other people is s-fold different than the infection fatality rate in the index person, then the cumulative risk of death of other people in the absence of vaccination of the index is

6 $${{{D}}}_{{{{\mathrm{other,}}}}\;{{{\mathrm{no}}}}\;{{{\mathrm{vaccination}}}}} = {{{\mathrm{ARfs}}}}$$

With similar considerations as above, after vaccination the probability of infection of the index becomes *A*(1 − *E*) and in the presence of risk compensation the probability of infection of the index becomes (1 − *E*)(1 − (1 − *A*)^*X*^). Under the assumption that the vaccination and risk compensation do not change the infection fatality rate of others who might be infected and *R* remains similar in the short-term in the same environment where the index person and others operate, then the cumulative risk of death of other people after vaccination of the index and accounting for risk compensation becomes

7 $${{{D}}}_{{{{\mathrm{other,}}}}\;{{{\mathrm{vaccination}}}}} = \left( {1 - {{{E}}}} \right)\left( {1 - \left( {1 - {{{A}}}} \right)^{{{X}}}} \right){{{\mathrm{Rfs}}}}$$

The net benefit for the other people is

8 $${{{\mathrm{Benefit}}}}\left( {{{{\mathrm{other}}}}} \right) = {{{D}}}_{{{{\mathrm{other,}}}}\;{{{\mathrm{no}}}}\;{{{\mathrm{vaccination}}}}} - {{{D}}}_{{{{\mathrm{other,}}}}\;{{{\mathrm{vaccination}}}}} = {{{\mathrm{ARfs}}}} - \left( {1 - {{{E}}}} \right)\left( {1 - \left( {1 - {{{A}}}} \right)^{{{X}}}} \right){{{\mathrm{Rfs}}}}$$

For this benefit to be eliminated because of risk compensation, one needs to have

ARfs − (1 − *E*)(1 − (1 − *A*)^*X*^)Rfs = 0, and solving for *X*, this becomes

9 $${{{X}}}\left( {{{{\mathrm{other,}}}}\;{{{\mathrm{eliminated}}}}\;{{{\mathrm{benefit}}}}} \right) = {{{\mathrm{log}}}}\left[ {\left( {1 - {{{A}}} - {{{E}}}} \right)/\left( {1 - {{{\mathrm{E}}}}} \right)} \right]/{{{\mathrm{log}}}}\left( {1 - {{{A}}}} \right)$$

For the benefit to be halved because of risk compensation, one needs to have

2(ARfs − (1 − *E*)(1 − (1 − *A*)]^*X*^)Rfs) = ARfs − (1 − *E*)ARfs, and solving for *X* this becomes

10 $${{{X}}}\left( {{{{\mathrm{other,}}}}\;{{{\mathrm{halved}}}}\;{{{\mathrm{benefit}}}}} \right) = {{{\mathrm{log}}}}\left[ {\left( {2 + {{{\mathrm{AE}}}} - 2{{{A}}} - 2{{{E}}}} \right)/2\left( {1 - {{{E}}}} \right)} \right]/{{{\mathrm{log}}}}\left( {1 - {{{A}}}} \right)$$

Of note, when *H* = 1, in the presented framework both the benefit for the index vaccinated person and for the other people are eliminated (or halved) with the same amount of risk compensation. In fact, in a setting where other people are also vaccinated, if they do become infected, they may have the same *H* as the vaccinated index person anyhow. To simplify matters, further calculations assume that *H* = 1 in the main analyses. Also of note, as more of the other people also get vaccinated, *R* would decrease accordingly.

### Numerical examples

Based on these considerations, the results presented explore the required values of risk compensation *X* that would diminish the personal, other or total benefit to half or to zero, for a range of values for the other parameters. This includes different values of vaccine efficacy *E* (low 20%, moderate 60%, high 80%, very high 95%), and different values of *A* (0.001–50%). The impact of modification of infection fatality rate by vaccination *H* (none *H* = 1, doubled *H* = 2, halved *H* = 0.5) is also explored.

### Reporting summary

Further information on research design is available in the Nature Research Reporting Summary linked to this article.

## Supplementary information

Reporting Summary

## Data Availability

No empirical data are included in the manuscript simulations.
